# Whey Protein Concentrate WPC-80 Improves Antioxidant Defense Systems in the Salivary Glands of 14-Month Wistar Rats

**DOI:** 10.3390/nu10060782

**Published:** 2018-06-17

**Authors:** Mateusz Falkowski, Mateusz Maciejczyk, Tomasz Koprowicz, Bożena Mikołuć, Anna Milewska, Anna Zalewska, Halina Car

**Affiliations:** 1 Department of Experimental Pharmacology, Medical University of Bialystok, 37 Szpitalna Street, 15-767 Bialystok, Poland; mateuszjanfalkowski@gmail.com (M.F.); koperchemik@poczta.onet.pl (T.K.); halina.car@umb.edu.pl (H.C.); 2 ZOZ Clinic of Dentistry and Medicine, E. and B. Falkowscy CLP., 17B Zarzecze Street, 16-300 Augustów, Poland; 3 Department of Physiology, Medical University of Bialystok, 2c Mickiewicza Street, 15-233 Bialystok, Poland; 4 Servier Polska LTD, 10 Jana Kazimierza Street, 01-248 Warszawa, Poland; 5 Department of Pediatrics, Rheumatology, Immunology and Metabolic Bone Diseases, Medical University of Bialystok, 17 Waszyngtona Street, 15-274 Białystok, Poland; bozenam@mp.pl; 6 Department of Statistics and Medical Informatics, Medical University of Bialystok, 37 Szpitalna Street, 15-767 Bialystok, Poland; anna.milewska@umb.edu.pl; 7 Department of Conservative Dentistry, Medical University of Bialystok, 24a M. Sklodowskiej-Curie Street, 15-274 Bialystok, Poland; azalewska426@gmail.com

**Keywords:** oxidative stress, salivary glands, aging, whey, salivary antioxidants

## Abstract

Whey protein concentrate (WPC) is characterized by powerful antioxidant properties, but its effect on redox homeostasis of salivary glands of aging organisms is still unknown. In this study, we are the first to evaluate the antioxidant barrier of salivary glands of 14-month Wistar rats fed WPC-80. Total antioxidant status (TAS), total oxidant status (TOS), oxidative stress index (OSI), activities of glutathione peroxidase (GPx), superoxide dismutase (SOD), catalase (CAT) as well as concentrations of reduced glutathione (GSH) are estimated in the submandibular and parotid glands of rats administered WPC-80 intragastrically for a period of 7 and 14 days. We demonstrate a significant increase in GSH, GPx and SOD in the salivary glands of rats fed WPC-80 for 14 days and a significant increase in TAS, GPx and SOD in the parotid glands of rats fed WPC-80 for 7 days compared to control rats. The beneficial effects of WPC-80 on salivary glands are also demonstrated by lower TOS and OSI in the parotid glands of rats fed WPC-80 compared to the submandibular glands. In summary, we demonstrate that WPC-80 improves redox homeostasis in salivary glands, particularly in the parotid glands of old rats.

## 1. Introduction

Aging is a complex biological process which results in damage to cell components, and progressive organ and system insufficiency [1]. Clinical consequences of aging within the oral cavity have recently become the focus of attention [2]. The changes concern mainly impairment of the salivary gland secretory function and the ensuing qualitative and quantitative changes in saliva [2,3]. Therefore, it is not surprising that older individuals complain very frequently of the subjective sensation of dry mouth (xerostomia), burning mouth syndrome (BMS) and an increased incidence of fungal infections and atrophic mucositis [3,4,5]. These conditions significantly impair the quality of life of older individuals and may cause other oral diseases. Impairment of the salivary gland function as a result of aging is found in as many as half of the world’s population of older individuals [3].

The aging process is influenced by a large number of factors, although oxidative stress is considered to play a key role in it [1,6]. It is believed that the aging process is crucially dependent on the enzymatic [glutathione peroxidase (GPx), superoxide dismutase (SOD), catalase (CAT)] and non-enzymatic antioxidative defense mechanisms [reduced glutathione (GSH), vitamin A and E], protecting living organisms from excessive reactive oxygen species (ROS) activity. It has been suggested that antioxidant supplementation could slow down organ changes associated with the aging process by eliminating the destructive impact of ROS on DNA, proteins and lipids and averting oxidative damage accumulation in cells. However, there are no effective therapeutic methods eliminating or preventing the bothersome complications associated with aging within the oral cavity [2,7,8]. Therefore, new substances which could effectively counteract impaired antioxidant defense mechanisms in old organisms are sought. One of such substances is whey protein, in particular, whey protein concentrate (WPC)-80 containing 80% of whey protein. Whey protein and its concentrates constitute a rich source of sulphur-containing amino acids (cysteines and methionines), which are important substrates in the biosynthesis of GSH [9,10,11,12]. GSH is one of the key cellular antioxidants, which also exhibits detoxifying properties, stimulates leukocyte proliferation and activity, and modulates immune functions [13]. Furthermore, thanks to the presence of numerous proteins, amino acids and growth factors, whey exhibits anticarcinogenic, hypotensive, antibacterial and antiviral activities [9,10,11,12].

We assumed that WPC-80 could impact on the enzymatic and non-enzymatic antioxidant systems of salivary glands, thus reducing the degree of their damage caused by oxidative stress. Therefore, the aim of our study was to evaluate the antioxidative barrier of parotid and submandibular glands of old rats fed WPC-80.

## 2. Materials and Methods

### 2.1. WPC-80 Composition

WPC-80 was produced by Dairy Cooperative in Mońki, Poland. WPC-80 was analyzed in the accredited laboratory from SJ Hamilton Poland LTD (Gdynia, Poland) and Rtech laboratory from Land O’Lakes Laboratories (St. Paul, MN, USA). The analyses included the assessment of the proteins, amino acids, fatty acids, vitamins, as well as minerals content. The humidity and calorific values of WPC-80 were also evaluated.

### 2.2. Animals

The study protocol was approved by the Local Animal Ethics Committee (permission numbers 60/2009, 12/2011 and 26/2013). The study was conducted on 14-month male Wistar rats (*n* = 40) with the initial body weight of 415–480 g. The animals were housed individually in separately ventilated cages (55 *×* 40 *×* 20 cm) at the Centre for Experimental Medicine, Medical University of Bialystok, Poland. Constant environmental conditions, i.e., temperature of 20 ± 2 °C, relative humidity of 65% and the 12-h light/dark cycle, were maintained throughout the experiment. The animals received standard rat pelleted feed containing 36% protein, 54% carbohydrates and 10% fat (Hasteller Company, Germany). Permanent, unrestricted access to food and drinking water was provided for the duration of the experiment. After a week’s adaptation period, the animals were randomly divided into 4 groups with 10 animals in each group (*n* = 10):

**C7**—a control group receiving a saline solution intragastrically for 7 days;
**C14**—a control group receiving a saline solution intragastrically for 14 days;
**WPC7**—an experimental group receiving WPC-80 intragastrically at the dose of 0.3 mg/kg BW for 7 days and;
**WPC14**—an experimental group receiving WPC-80 intragastrically at the dose of 0.3 mg/kg BW for 14 days.

Whey doses were determined based on literature analysis, and the most frequently used doses were selected [10,14].

Every day between 8:00 and 10:00 a.m., the appropriate amount of WPC-80 was dissolved in 0.5 mL of a 0.9% NaCl solution (saline), and after short immobilization of rats, it was administered intragastrically by an experienced researcher. At the same time, control rats received 0.5 mL of the saline solution intragastrically. After the experiment, rats were fasted overnight and anesthetized intraperitoneally with ketamine (80 mg/kg BW) and xylazine (5 mg/kg BW). All animals were bled by taking blood, and subsequently, submandibular and parotid glands were collected. Tissues were frozen in liquid nitrogen and stored at −80 °C until assayed. The blood was collected for a test tube with EDTA. Complete blood counts (HCT, hematocrit; HGB, hemoglobin; MCV, mean corpuscular volume; MCH, mean corpuscular hemaglobin; MCHC, mean corpuscular hemoglobin concentration; RBC, erythrocytes; PLT, platelets; WBC, leukocytes) were analyzed in the whole blood. Immediately after centrifugation (1700 rpm for 15 min, 4 °C), the obtained plasma was tested for biochemical parameters (ALT, alanine aminotransferase; AST, aspartate aminotransferase; amylase, albumin, creatinine, urea and uric acid). All determinations were performed using ABX Pentra 400 (Horiba, UK).

### 2.3. Homogenization

On the day of biochemical determinations, tissues were slowly thawed at 4 °C. The submandibular and parotid glands were weighed (OHAUS, Nanikon, Switzerland) and homogenized (1:10) on ice in an ice-cold potassium phosphate buffer (50 mM, pH 7.4) with a glass homogenizer (Schuett Homgen, Gottingen, Germany) [8]. To prevent oxidation of the sample during processing and storage, 10 μL of 0.5 M butylated hydroxytoluene (BHT; Sigma-Aldrich, Steinheim, Germany) in acetonitrile (ACN; Merck KGaA, Darmstadt, Germany) was added per 1 mL of tissue homogenates [8,15]. The resulting suspensions were divided into two parts: one half was used to determine the activity of GPx and SOD and centrifuged at 20,000*×* g (4 °C, 30 min; MPW-350R, Medical Instruments, Warsaw, Poland), while the other half was used for the determination of total antioxidant status (TAS), total oxidant status (TOS), OSI, CAT, GSH and total protein and centrifuged at 700*×* g (4 °C, 20 min). After centrifugation, the supernatants were collected and used for further redox assays.

### 2.4. Biochemical Assays

TAS, TOS, activities of GPx, SOD, CAT, as well as concentrations of GSH and total protein were estimated in the submandibular and parotid glands of rats administered WPC-80 intragastrically for a period of 7 and 14 days as well as control rats. Absorbance was measured with the spectrophotometer, Multiskan^TM^ Go (Thermo Scientific, Vantaa, Finland). All determinations were performed in duplicate samples and converted to milligram of the total protein.

### 2.5. Total Antioxidant/Oxidant Status

TAS was determined using the ImAnOx (TAS/TAC) Kit (Immundiagnostik, Bensheim, Germany). TAS measurement is based on the reaction between antioxidants present in a given sample and a measured quantity of hydrogen peroxide (H_2_O_2_). Nonactivated H_2_O_2_ is then measured calorimetrically by utilizing the formation of a colored complex by H_2_O_2_ and 5-thio-2-nitrobenzoic acid (TNB), of which maximum absorbance occurs at 450 nm.

TOS was determined using the PerOx (TOS/TOC) Kit (Immundiagnostik, Bensheim, Germany). The method is based on the reaction between peroxidase and peroxides present in a given sample, which results in the conversion of TNB to a colored product with the maximum absorbance at 450 nm.

Oxidative stress index (OSI) was calculated using the formula: OSI = TOS/TAS *×* 100 [16].

### 2.6. Enzymatic and Non-Enzymatic Antioxidants

GPx activity was determined using the commercial Bioxytech^®^ GPx-340 ^TM^ Kit (Oxis Research^TM^; Foster City, CA, USA). The determination of GPx activity is based on the reaction between oxidized glutathione (GSSG) and glutathione reductase (GR) which causes a reduction of GSSG to GSH. This stage co-occurs with the oxidation of NADPH to NADP^+^, which is connected with a decrease in absorbance at 340 nm. The supernatant of the salivary gland was added to a solution containing GSH, GR and NADPH. The enzyme reaction was initiated by adding the substrate (organic peroxide), tert-butyl hydroperoxide, and the absorbance was recorded at 340 nm.

SOD activity was determined using the Superoxide Dismutase Assay Kit (Cayman Chemical Company; Ann Arbor, MI, USA) and utilizing the reaction between the superoxide anions generated by xanthine oxidase and hypoxanthine with a tetrazolium salt. Absorbance changes were monitored at the wavelength of 450 nm.

CAT activity was determined using the spectrophotometric method devised by Aebi [17]. The method is based on measuring the speed of hydrogen peroxide decomposition at the wavelength of 240 nm.

GSH concentration was determined using the Glutathione Assay Kit (Cayman Chemical Company; Ann Arbor, MI, USA). The sulfhydryl group of GSH reacted with 5,5′-dithio-bis-2-nitrobenzoic acid (DTNB) and produces TNB. At the same time, GSH reacts with TNB and gives GSTNB (mixed disulfide), which is reduced by GR to recycle the GSH and produce more TNB. The rate of TNB formation is directly proportional to this recycling reaction, which is in turn proportional to the GSH level in the analyzed sample.

Total protein concentration was determined colorimetrically using the bicinchoninic acid assay (Thermo Scientific PIERCE BCA Protein Assay Kit, Rockford, IL, USA) with bovine serum albumin (BSA) as a standard. The method is based on the peptide bond ability to link with copper ions in a studied sample (II) in an alkaline environment. The complexes formed are then determined spectrophotometrically at 760 nm. The protein concentration, according to the manufacturer’s instructions, was expressed as milligram protein per 1 mL of tissue homogenate.

### 2.7. Stiatistical Analysis

Statistical analysis was performed using Statistica 12.0 version (StatSoft, Tulsa, OK, USA). In the case of data not following a normal distribution, the Mann–Whitney test and Spearman correlation were used in this study, and the results were presented as a bar chart with the individual data points. If data followed a normal distribution, a Student’s *t*-test was used and the results were expressed as mean ± SD.

## 3. Results

### 3.1. WPC-80 Composition

The conducted analyses demonstrated that proteins are the main component of WPC-80 and they constitute 77.6% of the concentrate’s mass. Additionally, carbohydrates, lipids, dietary fiber and other substances (ash, vitamins) were demonstrated to be present in WPC-80. The calorie content of the concentrate is 400 kcal/100 g WPC-80 (Supplementary Material).

### 3.2. Animal Characteristics

The final body mass was significantly higher in rats from WPC7 and WPC14 groups as compared to the control rats (Table 1). However, no significant differences were found in the biochemical (Table 2) and blood count parameters (Table 3) between the plasma of rats from different study groups.

**Figure 1:**
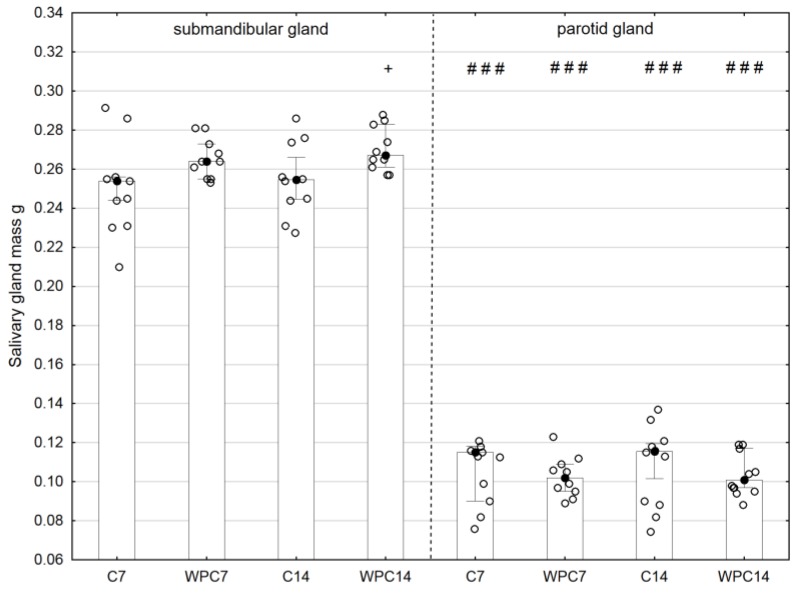
Salivary gland mass of rats from control and experimental groups. Abbreviations: C7, C14, control groups; WPC7, WPC14, experimental groups treated WPC-80. Data are presented as a bar chart with the individual data points; ^+^ *p* < 0.05 of the C14 submandibular gland and ### *p* < 0.001 of the submandibular gland, respectively, for the same groups. Because the results were not normally distributed, the Mann–Whitney test was used to compare differences between the studied groups.

A significantly higher submandibular gland mass was demonstrated after 14 days of WPC-80 administration in comparison with control rats (*p* < 0.05). The parotid gland mass of rats in all study groups was significantly lower than that of submandibular glands (*p* < 0.001), regardless of experiment duration (Figure 1).

**Figure 2:**
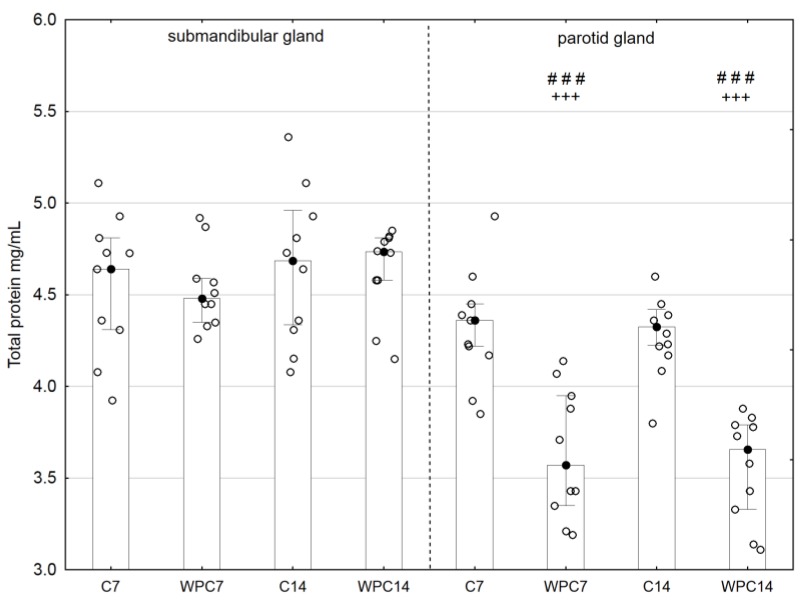
Total protein concentration of the submandibular and parotid glands of rats from control and experimental groups. Abbreviations: C7, C14, control groups; WPC7, WPC14, experimental groups treated WPC-80. Data are presented as a bar chart with the individual data points; ^+++^ *p* < 0.001 of the C14 submandibular gland and ^###^ *p* < 0.001 of the submandibular gland, respectively, for the same groups. Because the results were not normally distributed, the Mann-Whitney test was used to compare differences between the studied groups.

Exposure to WPC-80 for 7 and 14 days resulted in a statistically lower total protein content in the parotid glands in comparison with the corresponding controls (*p* < 0.001) and in comparison to the submandibular glands (*p* < 0.001) (Figure 2).

### 3.3. Total Antioxidant/Oxidant Status

**Figure 3:**
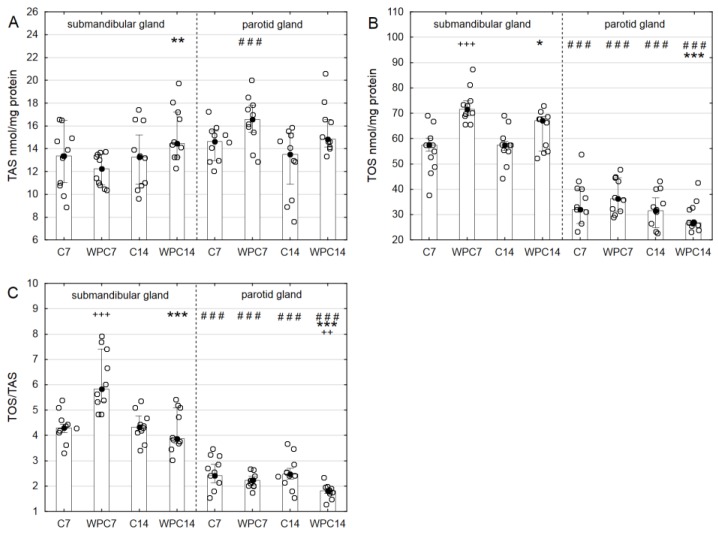
Total antioxidant status (**A**), total oxidant status (**B**) and oxidative stress index (**C**) in the salivary glands of rats from control and experimental groups. Abbreviations: C7, C14, control groups; WPC7, WPC14, experimental groups treated WPC-80; OSI, oxidative stress index; TAS, total antioxidant status; TOS, total oxidant status. Data are presented as a bar chart with the individual data points; ^+++^ *p* < 0.001 of the C7 submandibular gland, *p < 0.05 of the WPC7 submandibular gland, ** *p* < 0.01 of the WPC7 submandibular gland, *** *p* < 0.001 of the WPC7 parotid gland and ^###^ *p* < 0.001 of the submandibular gland, respectively, for the same groups. Because the results were not normally distributed, the Mann–Whitney test was used to compare differences between the studied groups.

A significant increase in TAS in the submandibular glands of animals receiving WPC-80 for 14 days in comparison to the group receiving WPC-80 for the period of 7 days (*p* < 0.01) was observed. TAS was significantly enhanced in the parotid glands of rats fed WPC-80 for 7 days in comparison to the control group (*p* < 0.01). A significantly higher level of TAS was observed in the parotid glands of rats receiving WPC-80 for 7 days in comparison to the submandibular glands (*p* < 0.001) (Figure 3A).

TOS levels were significantly higher in the submandibular glands of animals receiving WPC-80 for 7 days in comparison to the control group (*p* < 0.001). Extending the duration of WPC-80 administration resulted in a significant decrease in TOS in the submandibular glands of rats in comparison to the group receiving WPC-80 for 7 days (*p* < 0.05). TOS levels in the parotid glands of animals receiving WPC-80 for 7 days did not change, while the administration of WPC-80 for 14 days caused a significant decrease in TOS in the parotid glands of rats in comparison to the group receiving WPC-80 for 7 days (*p* < 0.001). A significantly decreased level of TOS was observed in the parotid glands in comparison to the submandibular glands of the studied and control animals (*p* < 0.001) (Figure 3B).

A significantly higher OSI (TOS/TAS ratio) was observed in the submandibular glands of animals receiving WPC-80 for 7 days in comparison to the control group (*p* < 0.001). Extending the duration of WPC-80 administration to 14 days resulted in a statistically significant decrease in OSI in the parotid glands in relation to the group receiving WPC-80 for 7 days (*p* < 0.001). A significantly decreased OSI was also observed in the parotid glands of animals receiving WPC-80 for 14 days in comparison to the control group (*p* < 0.01) and in comparison to the group receiving WPC-80 for 7 days (*p* < 0.001). Furthermore, the OSI in the parotid glands of the control group, following the administration of WPC-80 for 7 and 14 days, was significantly reduced in comparison to the values observed in the submandibular glands of those animals (*p* < 0.001) (Figure 3C).

### 3.4. Enzymatic and Non-Enzymatic Antioxidants

**Figure 4:**
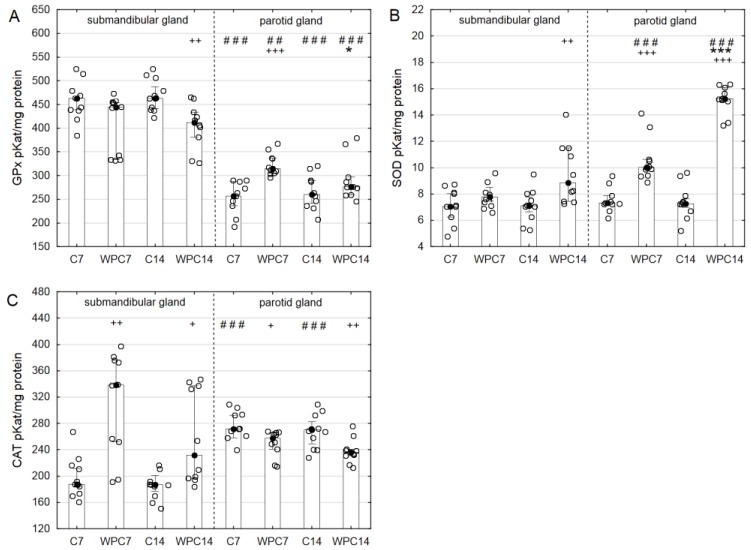
Glutathione peroxidase (**A**), superoxide dismutase (**B**) and catalase (**C**) activities in the salivary glands of rats from control and experimental groups. Abbreviations: C7, C14, control groups; WPC7, WPC14, experimental groups treated WPC-80; CAT, catalase; GPx, glutathione peroxidase; SOD, superoxide dismutase. Data are presented as a bar chart with the individual data points; ^+^ *p* < 0.05 of the C14 submandibular gland or of the C7 parotid gland, ^++^ *p* < 0.01 of the C14 submandibular gland, ^+++^ *p* < 0.001 of the C7 parotid gland, * *p* < 0.05 of the WPC14 parotid gland, *** *p* < 0.001 of the WPC14 parotid gland, and ^##^ *p* < 0.01 and ^###^ *p* < 0.001 of the submandibular gland, respectively, for the same groups. Because the results were not normally distributed, the Mann–Whitney test was used to compare differences between the studied groups.

The administration of WPC-80 for 14 days resulted in a statistically significant higher GPx activity in the submandibular glands in comparison to the parotid glands (*p* < 0.01). GPx activity in the parotid glands was significantly elevated in the group of animals fed WPC-80 for 14 days in comparison to the relevant control group (*p* < 0.001) (Figure 4A).

A significantly higher SOD activity in the parotid glands of rats receiving WPC-80 for 7 and 14 days in comparison to the relevant control group (*p* < 0.001) was observed. Animals administered whey displayed significantly an enhanced SOD activity in the parotid glands in comparison to the submandibular glands throughout the 7-day as well the 14-day duration of WPC-80 administration (Figure 4B).

After WPC-80 administration for 7 days (*p* < 0.01) and 14 days (*p* < 0.05), a significantly higher CAT activity in the submandibular glands was observed in comparison to the relevant control groups. A significantly reduced CAT activity was observed in the parotid glands of rats after the administration of WPC-80 for 7 days (*p* < 0.05) and 14 days (*p* < 0.01) in comparison to the relevant control groups. A significantly increased CAT activity was observed in the parotid glands as compared to the CAT activity in the submandibular glands of control rats receiving WPC-80 for 7 and 14 days (*p* < 0.001) (Figure 4C).

**Figure 5:**
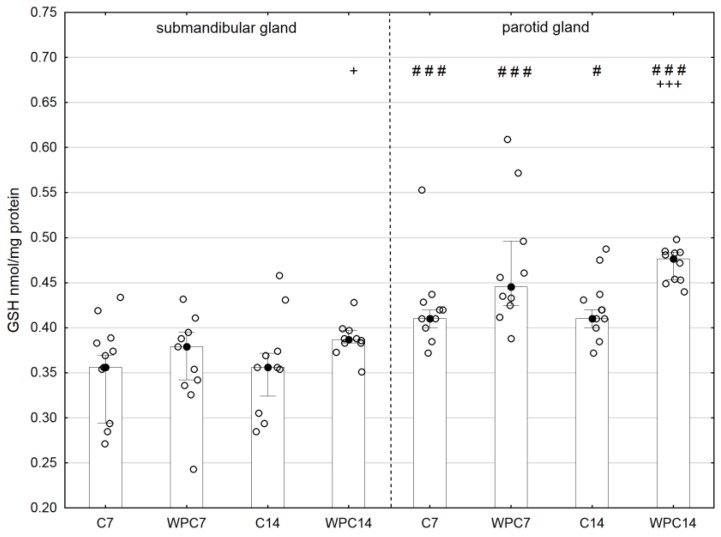
Reduced glutathione (GSH) content in the salivary glands of rats from control and experimental groups. Abbreviations: C7, C14, control groups; WPC7, WPC14, experimental groups treated WPC-80; GPx, glutathione peroxidase. Data are presented as a bar chart with the individual data points; ^+^ *p* < 0.05 of the C14 submandibular gland or of the C7 parotid gland; ^+++^ *p*< 0.001 of the C14 parotid gland, and ^#^ *p* < 0.05 and ^###^ *p* < 0.001 of the submandibular gland, respectively, for the same groups. Because the results were not normally distributed, the Mann–Whitney test was used to compare differences between the studied groups.

A significantly higher GSH concentration was observed in the submandibular glands of animals receiving WPC-80 for 14 days than that for 14-day controls (*p* < 0.05). The GSH concentration in the parotid glands of rats receiving WPC-80 for 7 days did not change significantly, whereas extending exposure to WPC-80 to 14 days resulted in a statistically significant increase in the tested parameter in comparison to the values in 14-day controls (*p* < 0.001). Significantly enhanced GSH concentrations were observed in the parotid glands in comparison to the submandibular glands in both the control groups (*p* < 0.001–7-day controls and *p* < 0.05–14-day controls) and groups receiving WPC-80 (*p* < 0.001) (Figure 5).

### 3.5. Correlations

The results of statistically significant correlations are presented in Table 4. Interestingly, positive correlations between TAS levels, CAT activities, and GSH concentrations was found in the parotid glands of rats fed WPC-80 for 7 days. OSI correlated negatively with GSH concentrations and positively with TOS levels in the parotid glands of rats receiving WPC-80 for 14 days (Table 4).

## 4. Discussion

This is the first study, in which the effect of WPC-80 on redox homeostasis in salivary glands has been evaluated. It was demonstrated that WPC-80 affects the salivary redox balance and generally stimulates antioxidant defense mechanisms.

Oxidative stress is one of the most important, and at the same time, one of the most common pathological factors responsible for the induction and progression of the majority of contemporary diseases and pathological states [18,19,20]. The process is defined as a disturbance in the balance between ROS production and antioxidant defenses, which leads to oxidative damage of cellular components. Oxidative stress is thereby responsible for the structural and functional impairment of a number of organs and systems [18,20], including the salivary glands [21,22,23,24]. Furthermore, it is believed that oxidative stress leads to salivary gland damage associated with aging [3]. The presence of anatomical and morphological changes has been demonstrated in salivary gland parenchyma of older individuals, which leads to impaired salivary flow, a decrease in its buffering capacity, the weakening of immune response mechanisms and disturbances in the qualitative composition of saliva, which may result from the cellular accumulation of DNA, protein and lipid oxidation products [3,25,26]. With advancing age, a significant reduction in the effectiveness of enzymatic and non-enzymatic antioxidant defense systems also occurs [25,26]. Therefore, of interest are substances capable of slowing down the aging process, such as compounds displaying antioxidant activities, including whey.

The qualitative and quantitative assessment of oxidative stress is conducted using a number of biomarkers which include the detection of ROS activity, measurement of the levels of individual antioxidants and also the evaluation of damage caused by ROS and their derivatives [27]. However, the total antioxidant effect is not an aggregate of concentrations/activity of individual antioxidants [28]. An exceptionally useful marker utilized in the assessment of oxidative stress intensity is TAS [28,29]. It is believed that TAS reflects the efficiency of both enzymatic and non-enzymatic antioxidant defense mechanisms in the body. In the present study, we demonstrated a significantly higher level of TAS after a 7-day period of supplementation in the parotid glands of rats fed WPC in comparison with the controls and the submandibular glands. The beneficial impact of WPC-80 on the salivary gland activity was also reflected by TOS and OSI, which were significantly lower in the parotid glands of rats fed whey for 7 and 14 days than that in the submandibular glands of the same groups. It should be remembered that OSI expresses an objective relationship between the efficiency of antioxidant mechanisms (TAS) and oxidant levels (TOS), and therefore, the obtained results suggest that a whey diet improves the main antioxidant properties of parotid glands. The presented results are not surprising, due to the fact that in physiological conditions, the parotid glands are the primary source of salivary antioxidants [16]. These observations were confirmed by a significantly higher SOD and GPx activity in the parotid glands of rats fed WPC-80 for 7 and 14 days (in comparison to the submandibular glands and to the relevant controls). We also observed that extending the duration of WPC-80 administration significantly reduced TOS and OSI levels in both glands (↓TOS, ↓OSI in the parotid glands of rats fed WPC-80 for 14 days in contrast to those for 7 days).

However, the increased TOS and OSI levels in the submandibular gland observed in the first week of WPC-80 administration may indicate an increased production of free radicals under the high-protein supplementation. As shown in other studies, excessive supply of dietary protein may cause oxidative stress in the salivary glands of high-protein fed rats [7,30]. It is well known that the higher amount of dietary proteins cannot be stored in the body, resulting in their enhanced decomposition and elevated free radical production in the mitochondrial respiratory chain [30,31]. Therefore, the early changes in the redox homeostasis observed in our study may suggest a physiological response to the increased whey-proteins administration. This can also be demonstrated by the differences in CAT and GPx specific activities. As shown in other studies, the parotid gland is more prone to the free radical damage in these conditions [30]. However, in this research, the animals received standard laboratory rat chow, and WPC-80 was administered only in the form of intragastric supplementation (but not as the primary source of food). Additionally, unlike most other proteins, whey has a significant antioxidant potential and may increase production of the most important salivary antioxidant—GSH [32]. Indeed, we demonstrated significantly enhanced GSH concentrations in both pairs of glands of rats fed WPC-80 for 14 days in comparison to controls and significantly higher GSH levels in the parotid glands of rats fed whey for 7 and 14 days in comparison to the submandibular glands. These results confirm the ability of WPC-80 to induce GSH biosynthesis in the salivary glands of old rats. The result can be explained by the presence of a considerable number of sulfur-containing amino acids: methionine and cysteine in whey proteins which enhance the endogenous production of glutathione in the γ-glutamyl cycle. The principal substrate for glutathione synthesis in this process, in addition to glutamate and glycine, is cysteine found in whey [33]. It appears that an increase in the GSH concentration following WPC-80 administration may protect salivary glands from excessive ROS production in aging organisms, as well as participate in the regeneration of other salivary antioxidants (mainly Vitamins C and E) [7,32]. Furthermore, GSH plays an important role in the regeneration of oxidatively damaged DNA, proteins and lipids, and also keeps protein thiol groups in a reduced state, thereby conditioning their functional and enzymatic activities, and thus slowing down the aging process [25,34].

There is a lack of studies evaluating the impact of whey on the salivary gland function in the current literature. In one of the few experiments, the protective effect of whey protein isolate (WPI) on the damaging effect of the anabolic steroid nandrolone within the parotid glands of rats has been demonstrated [35]. The histological structure and ultrastructure of the salivary glands of animals fed WPI for 3 months did not display significant morphological changes in comparison to the controls—the normal structure and ultrastructure of salivary gland parenchyma were preserved, except for marginal vasodilatation. This suggests that a high-protein whey diet does not condition pathological changes in the glandular tissue of salivary glands, and can, via a mild dilatory effect on blood vessels, increase the volume of secreted saliva, and in effect, enhance treatment efficacy of conditions causing xerostomia [35]. 

One of the parameters characterizing the salivary gland secretory capacity is the total protein concentration [2,36]. In our study, we did not notice significant differences in the total protein concentration in the submandibular glands of rats fed WPC-80. However, we demonstrated a significantly lower total protein concentration (irrespective of the duration of WPC-80 administration) in the parotid glands of rats fed WPC-80 in comparison to the controls. The obtained results may indicate disturbances in the synthesis and/or secretion of proteins by the parotid glands of old rats in response to WPC-80 administration [23,36]. Although our study did not assess the animal’s food intake, it can be assumed that the decrease in total protein content may be due to the smaller amount of food consumed by rats receiving a high-protein diet, which is consistent with the results of other studies [30,31,37]. It has been shown that reduced food intake may result in the impaired exocytosis and protein synthesis only in the parotid glands (not in the submandibular glands) [30,38]. Additionally, histological research conducted to date has demonstrated that the parotid glands of rats fed a high-protein diet are characterized by a reduction in the size of acinous structures and efferent ducts, while the ultrastructure of submandibular glands is maintained [39]. The elucidation of these alterations requires further research and observations. In particular, histochemical studies would be helpful to explain the nature of the observed changes.

Whey is characterized by nutritious, hypotensive, anticancer, antimicrobial and immunomodulatory properties, although it is distinguished by its significant antioxidant potential [9,10,11,12]. The results of our study point to the conclusion that a whey-rich diet modulates the salivary antioxidant systems and generally improves salivary gland redox properties, in particular those of the parotid glands. The positive total antioxidant effect of a whey-rich diet is demonstrated by the positive correlation between GSH and TAS levels in the parotid glands of rats fed WPC-80 for 7 days and the negative correlation between GSH and OSI concentrations in rats administered WPC-80 for 14 days. We believe that the enhancement of the salivary gland antioxidant capacity of rats fed a whey-rich diet may improve the redox balance not only within the oral cavity itself, but also within the whole body. Enzymatic and non-enzymatic salivary antioxidants constitute a type of barrier protecting live organisms from the damaging effects of numerous exogenous factors, such as air pollution, drugs and other xenobiotics, as well as ionizing, ultraviolet and ultrasound radiation [2,21].

Not without significance is the impact of WPC-80 on general health of rats. We did not observe any statistically changes in the blood counts and plasma biochemical parameters between the WPC-80 and control rats. In WPC-80 groups, we only noticed the enhanced rat’s body mass, which may be due to the high caloric value of WPC used in the experiment. Therefore, higher protein supply in the diet may promote gain in muscle mass as well as attenuate the natural loss of muscle content related to aging [12,40]. It has been demonstrated that whey proteins induce muscle protein synthesis in a greater degree than other peptides (e.g., soy and casein) [40]. This fact is not surprising, because whey proteins are rich source of branched-chain amino acids.

When analyzing the results of our study, one should consider its limitations. We evaluated solely the concentration/activity of selected salivary antioxidants, and therefore the evaluation of different antioxidant defense markers may lead to dissimilar observations and conclusions. It should also be borne in mind that the BHT addition to tissue homogenates may affect the redox balance of the salivary glands. However, in order to thoroughly evaluate the impact of WPC-80 on salivary gland redox balance, the assessment of oxidative stress markers of DNA, proteins and lipids would be recommended. Only then would we be able to draw firm conclusions concerning the protective effect of WPC-80 on preventing salivary gland oxidative stress. However, our study is the first to document the antioxidant capacity of salivary glands of old rats and justifies the necessity for further research in this area.

## Figures and Tables

**Table 1:** Body weight of rats from control and experimental groups. Abbreviations: C7, C14, control groups; whey protein concentrate (WPC)7, WPC14, experimental groups treated WPC-80. Data are presented as median, minimum and maximum. Because the results were not normally distributed, the Mann–Whitney test was used to compare differences between the studied groups.

Groups	Initial body Weight (g)			Final Body Weight (g)		
	Median	Min	Max	Median	Min	Max
C7	450.0	415.0	485.0	470.0	436.0	520.0
C14	454.5	420.0	490.0	482.0	455.0	521.0
WPC7	449.0	420.0	484.0	522.5	449.0	557.0
WPC14	450.0	429.0	486.0	523.0	489.0	534.0
*p* value						
WPC7:C7	1.0			0.01		
WPC14:C14	1.0			0.01		

**Table 2:** Plasma biochemical parameters of rats from control and experimental groups. Abbreviations: ALT, alanine aminotransferase; AST, aspartate aminotransferase; C7, C14, control groups; WPC7, WPC14, experimental groups treated WPC-80. Data are presented as mean ± SD. Because the results were normally distributed, a Student’s *t*-test was used to compare differences between the studied groups.

Groups	ALT (U/L)	AST (U/L)	Amylase (U/L)	Albumin (µmol/L)	Uric Acid (µmol/L)	Urea (mmol/L)	Creatinine (mg/dL)
C7	53.2 ± 6.3	122.3 ± 23.4	1552.5 ± 129.9	424.9 ± 6.9	29 ± 4.6	7.13 ± 0.8	0.5 ± 0.07
C14	51.6 ± 8.8	120.4 ± 25.8	1522.3 ± 134.8	433.6 ± 3.7	28 ± 5.4	7.21 ± 0.4	0.5 ± 0.05
WPC7	48.7 ± 12.7	122.2 ± 34.9	1530.9 ± 157.1	427.9 ± 20.1	32 ± 13.8	7.39 ± 0.5	0.4 ± 0.04
WPC14	61.1 ± 13.5	103.5 ± 30.1	1423.6 ± 206.1	455.4 ± 12.7	35 ± 4.2	7.93 ± 0.6	0.5 ± 0.01
*p* value							
WPC7:C7	1.00	1.00	1.00	0.53	1.00	0.58	1.00
WPC14:C14	1.00	1.00	1.00	0.16	0.50	1.00	1.00

**Table 3:** Morphological parameters of rats from control and experimental groups. Abbreviations: C7, C14, control groups; WPC7, WPC14, experimental groups treated WPC-80; HCT, hematocrit; HGB, hemoglobin; MCV, mean corpuscular volume; MCH, mean corpuscular hemaglobin; MCHC, mean corpuscular hemoglobin concentration; RBC, erythrocytes; PLT, platelets; WBC, leukocytes. Data are presented as mean ± SD. Because the results were normally distributed, a student’s *t*-test was used to compare differences between the studied groups.

Groups	WBC (× 10^12^/L)	RBC (M/µ/L)	HGB (g/dL)	HCT (%)	PLT (x 10^9^/L)	MCV (fL)	MCH (pg)	MCHC (g/dL)
C7	3.1 ± 0.7	8.38 ± 0.2	14.7 ± 0.4	44.3 ± 1.9	673 ± 141	52 ± 0.6	18.0 ± 0.5	33.6 ± 0.5
C14	2.8 ± 0.9	8.14 ± 0.3	14.3 ± 0.4	43.3 ± 1.5	691 ± 73	53 ± 0.5	17.5 ± 0.3	32.5 ± 0.3
WPC7	2.1 ± 0.8	8.51 ± 0.4	14.4 ± 0.8	44.6 ± 2.2	691 ± 66	52 ± 0.8	17.7 ± 0.4	33.4 ± 0.5
WPC14	1.7 ± 0.4	8.48 ± 0.1	14.4 ± 0.3	43.8 ± 0.6	677 ± 31	53 ± 0.3	17.9 ± 0.3	33.5 ± 0.3
*p* value								
WPC7:C7	1.00	1.00	1.00	1.00	1.00	1.00	1.00	1.00
WPC14:C14	0.15	0.40	1.00	0.28	1.00	1.00	1.00	1.00

**Table 4:** Correlations between analyzed redox parameters in the submandibular and parotid glands of rats from control and experimental groups. Abbreviations: C7, C14, control groups; WPC7, WPC14, experimental groups treated WPC-80; CAT, catalase; GPx, glutathione peroxidase; GSH, reduced glutathione; SOD, superoxide dismutase; TAS, total antioxidant status; TOS, total oxidant status; OSI, oxidative stress index.

Pair of Variables	Group	Salivary Gland	*r*	*p*
GPx & SOD	C 7	submandibular	−0.79	0.03
GSH & SOD	C 7	parotid	0.69	0.03
GSH & SOD	C 14	parotid	−0.81	0.04
OSI & TOS	WPC 7	submandibular	0.74	0.01
TAS & GSH	WPC 7	parotid	0.65	0.04
TAS & CAT	WPC 7	parotid	0.93	<0.001
TAS & OSI	WPC 14	submandibular	−0.78	0.001
TAS & GPx	WPC 14	parotid	−0.65	0.04
OSI & GSH	WPC 14	parotid	−0.75	0.01
OSI & TOS	WPC 14	parotid	0.65	0.01

## Supplementary Materials

The following are available online at http://www.mdpi.com/2072-6643/10/6/782/s1.
