# Supplementary material for: Whey Protein Concentrate WPC-80 Improves Antioxidant Defense Systems in the Salivary Glands of 14-Month Wistar Rats
Source: Nutrients. 2018 Jun 17;10(6):782. doi: 10.3390/nu10060782 (PMC6024865; doi:10.3390/nu10060782)
Supplement: Supplementary file 1 [file nutrients-10-00782-s001.pdf]

**General composition**	**%**
Protein	77.6
Dietary fiber	<0.5
Carbohydrates	8.4
Ash	2.8
Fat	6.19
Humidity	5.02
The calorific value	1691 kJ/100g
**Protein**	**%**
β-lactoglobulin	39.96
α-lactalbumin	9.52
immunoglobulins	5.55
bovine serum albumin	3.17
**Endogenous amino acids**	**%**
aspartic acid	8.91
glutamic acid	15.01
serine	4.26
glycine	1.54
tyrosine	2.71
arginine	2.27
cysteine	1.56
alanine	4.14
proline	5.06
**Exogenous amino acids**	**%**
histidine	1.48
valine	5.19
methionine	1.83
threonine	5.81
isoleucine	4.79
leucine	9.09
phenylalanine	2.84
lysine	8.05
tryptophan	12.97 g/kg
**Fatty acids**	**g/100g fat**
butanoic acid (butyric acid) C4:0	1.8
hexanoic acid (caproic) C6:0	0.9
octanoic acid (caprylic) C8:0	0.8
decanoic acid (capric) C10:0	2.1
undecanoic acid C11:0	0.1
dodecanoic acid (lauric) C12:0	2.8
tridecanoic acid C13:0	< 0.1
tetradecanoic acid (myristic) C14:0	11.0
pentadecanoic acid C15:0	1.2
hexadecanoic acid (palmitic) C16:0	31.2
heptadecanoic acid C17:0	0.7
octadecanoic acid (stearic) C18:0	11.7
eicosanoic acid (arachidic) C20:0	0.2
docosanoic acid (behenic acid) C22:0	0.2
tetracosanoic acid C24:0	0.1
tetradecenoic acid C14:1	0.9
pentadecenoic acid C15:1	< 0.1
haxadecenoic acid (palmitoleic) C16:1	2.3
heptadecenoicacid C17:1	0.2
(Z) -9-Octadecenoic acid (Oleic) C18:1	26.4
eicosenoic acid(gadoleic) C20:1	<0.1
(Z) -13-docozaenowy acid C22:1	< 0.1
nervonic acid C24:1	< 0.1

(Z, Z) -9.12-octadecadienoic C18:2	2.2
octadecatrienoic acid C18:3	0.7
eicosadienoic acid C20:2 n-6	< 0.1
docosadienoic acid C22:2 n-6	< 0.1
other fatty acids	2.4
**Vitamins**	**per 100g**
Vitamin A (retinol)	20 μg
Vitamin B1 (thiamine)	0.11 mg
Vitamin B2 (riboflavin)	1.1 mg
Vitamin B6 (pyridoxine)	0.11 mg
Vitamin E (α-tocopherol)	0.2 mg
Vitamin B12	20.5 μg
Vitamin B9	564 μg
Vitamin D_3	<0.5 μg
Vitamin K_3	<1 mg/kg
**Minerals**	
sodium	0.15%
potassium	0.47%
calcium	0.47%
phosphorus	0.34%
magnesium	791 mg/kg
zinc	5.32 mg/kg
copper	0.63 mg/kg
iron	4.06 mg/kg

Table 1. Composition of WPC-80.
